# Neighborhood Cohesion, Loneliness, and Cognitive Function: Effects of Race and Place

**DOI:** 10.1093/geroni/igaf122.434

**Published:** 2025-12-31

**Authors:** Jeffrey Stokes, Kenzie Latham-Mintus, Heather Farmer, Setarreh Massizhadegan

**Affiliations:** University of Massachusetts Boston, Boston, Massachusetts, United States; Indiana University Indianapolis, Indianapolis, Indiana, United States; University of Delaware, Newark, Delaware, United States; University of Massachusetts Boston, Boston, Massachusetts, United States

## Abstract

Racial/ethnic disparities in cognition and dementia are well-established. Social determinants of health researchers have identified neighborhood context as one potential contributor to these disparities, though to date most research has focused on neighborhood built environment and objective neighborhood characteristics (e.g., neighborhood SES, racial/ethnic demographics). More recently, research on “cognability” has identified neighborhood social context as an important determinant of both well-being and cognitive health among the aging population. Neighborhood social cohesion, in particular, may help to reduce loneliness and thereby improve cognitive aging among midlife and older adults. However, access to high-quality and cohesive neighborhood settings varies by race/ethnicity in the U.S., as too may the association of neighborhood social cohesion with well-being outcomes such as loneliness. This study uses three-wave longitudinal data from the 2010-2020 waves of the Health and Retirement Study (HRS; N = 7,879) to examine whether (a) neighborhood social cohesion is associated with loneliness and, thereby, cognitive functioning over an 8-year period, as well as (b) whether this mediation model varies by race/ethnicity. Results indicate that non-Hispanic White adults reported the highest neighborhood social cohesion, the lowest loneliness, and exhibited the best cognitive functioning over the study period. Moreover, neighborhood social cohesion was associated with reduced loneliness only among non-Hispanic White participants. Although loneliness was associated with greater declines to cognition over time across all racial/ethnic groups, only non-Hispanic White participants’ cognitive trajectories benefited from living in socially cohesive neighborhoods, via reduced loneliness. We discuss implications of findings for health disparities research in light of the “cognability” framework.

